# Supplementary material for: An instrument for measuring job satisfaction (VIJS): A validation study for community pharmacists in the context of the COVID-19 pandemic in Vietnam
Source: PLoS One. 2022 Nov 4;17(11):e0276918. doi: 10.1371/journal.pone.0276918 (PMC9635709; doi:10.1371/journal.pone.0276918)
Supplement: S1 File — (DOCX) [file pone.0276918.s001.docx]

**An instrument for measuring job satisfaction of community pharmacists**

**English version**

Code:..............................................................

Day of data collection: ......../.........../...............

Dear pharmacists. The research team from Hanoi University of Pharmacy is conducting a survey in order to evaluate the job satisfaction of community pharmacists in the context of the COVID-19 pandemic in Vietnam. We truly appreciate your contribution and participation in our research. The time for answering questions is about 10 minutes. All information in this data collection form is only used for studying. The identity of drugstores and pharmacists will be kept confidential. We hope that you can answer questions as completely and honestly as possible. Sincerely thank.

Do you agree to take part in this survey?

🞎 Yes 🞎 No

**Pharmacists’ information**

1. Sex: 🞎 Male 🞎 Female

2. Year of birth: .........................................................................................................................................

3. Marital status: 🞎 Married 🞎 Not married

4. Highest level of education

🞎 Middle 🞎 College

🞎 University 🞎 Post-university (Master, PhD...)

5. Working experience in medicine outlets (years): ...................................................................................

7. Time of working per day (hours): ...........................................................................................................

8. Salary per month (million Vietnam dongs)

🞎 < 2 🞎 2 – less than 4

🞎 4 – 6 🞎 > 6

9. The average number of customers per day (people)

🞎 < 30 🞎 30 – less than 50

🞎 50 - 100 🞎 > 100

10. Phone number: ...................................................................................................................................

11. Medicine outlet’s address: ..................................................................................................................

|  | **Items** | **Strongly disagree** | **Disagree** | **Normal** | **Agree** | **Strongly agree** |
| --- | --- | --- | --- | --- | --- | --- |
| **A** | **Satisfied with physical working conditions** | | | | | |
| A1 | I am satisfied with the facilities of my medicine outlet. | 1 | 2 | 3 | 4 | 5 |
| A2 | My workplace is spacious, clean, and airy. | 1 | 2 | 3 | 4 | 5 |
| A3 | I am working in safe conditions. | 1 | 2 | 3 | 4 | 5 |
| A4 | My medicine outlet has full equipment for professional work (such as a computer with an Internet connection, an air conditioner...) | 1 | 2 | 3 | 4 | 5 |
| A5 | There are enough means to help prevent the spread of disease/pandemic in my medicine outlet (such as face masks, hand sanitizers...) | 1 | 2 | 3 | 4 | 5 |
| **B** | **Satisfied with work nature** | | | | | |
| B1 | I am satisfied with my current job position. | 1 | 2 | 3 | 4 | 5 |
| B2 | The work that I am doing is suitable for the qualifications and skills that I was trained. | 1 | 2 | 3 | 4 | 5 |
| B3 | I can use and promote my capacity well at work. | 1 | 2 | 3 | 4 | 5 |
| B4 | Professional work (such as selling medicines, and patient counseling) fulfills my career aspirations. | 1 | 2 | 3 | 4 | 5 |
| B5 | I can meet and interact with many people. | 1 | 2 | 3 | 4 | 5 |
| **C** | **Satisfied with income and other benefits** | | | | | |
| C1 | I am satisfied with my current income. | 1 | 2 | 3 | 4 | 5 |
| C2 | My salary is commensurate with my current job position. | 1 | 2 | 3 | 4 | 5 |
| C3 | The bonus and remuneration I received are commensurate with my work performance. | 1 | 2 | 3 | 4 | 5 |
| C4 | Additional income (bonus) is distributed equally. | 1 | 2 | 3 | 4 | 5 |
| **D** | **Satisfied with management policies and managers** | | | | | |
| D1 | The managers always care about the lives of employees. | 1 | 2 | 3 | 4 | 5 |
| D2 | The managers treat all employees in my medicine outlet fairly and suitably. | 1 | 2 | 3 | 4 | 5 |
| D3 | The managers have enough capacity and knowledge to effectively manage and monitor the work at the medicine outlet. | 1 | 2 | 3 | 4 | 5 |
| D4 | The managers trust me in my work. | 1 | 2 | 3 | 4 | 5 |
| D5 | The managers always support and guide all employees enthusiastically. | 1 | 2 | 3 | 4 | 5 |
| D6 | The managers always listen to opinions and quickly respond to employees. | 1 | 2 | 3 | 4 | 5 |
| D7 | The managers always praise/encourage employees when they do a good job. | 1 | 2 | 3 | 4 | 5 |
| **E** | **Satisfied with the relationships with coworkers and customers/patients** | | | | | |
| E1 | My coworkers are competent and knowledgeable. | 1 | 2 | 3 | 4 | 5 |
| E2 | My coworkers are hospitable and friendly people. | 1 | 2 | 3 | 4 | 5 |
| E3 | My coworkers and I usually share experiences and help each other in our work. | 1 | 2 | 3 | 4 | 5 |
| E4 | My coworkers are trustworthy. | 1 | 2 | 3 | 4 | 5 |
| E5 | My coworkers and I care for and help each other in life. | 1 | 2 | 3 | 4 | 5 |
| E6 | I am satisfied with my relationship with patients/customers. | 1 | 2 | 3 | 4 | 5 |
| E7 | Patients/customers have a respectful attitude towards me. | 1 | 2 | 3 | 4 | 5 |
| **F** | **Satisfied with learning and advancement opportunities** | | | | | |
| F1 | I am satisfied with the training and advancement opportunities at my outlet. | 1 | 2 | 3 | 4 | 5 |
| F2 | I have been trained in professional knowledge and skills regularly. | 1 | 2 | 3 | 4 | 5 |
| F3 | I have many opportunities to participate in professional training courses (such as clinical pharmacy, drug information, drug consulting and using...) | 1 | 2 | 3 | 4 | 5 |
| F4 | I will have many opportunities to advance to a higher position if I work hard. | 1 | 2 | 3 | 4 | 5 |
| F5 | I have opportunities to study and promote my capacity at work. | 1 | 2 | 3 | 4 | 5 |
| F6 | My medicine outlet has fair and clear promotion policies. | 1 | 2 | 3 | 4 | 5 |
| **O** | **The outcome** |  |  |  |  |  |
| O1 | Overall, I'm satisfied with my current job at my medicine outlet. | 1 | 2 | 3 | 4 | 5 |
| O2 | In general, I want to stick with my current job and my medicine outlet for a long time. | 1 | 2 | 3 | 4 | 5 |
| O3 | If I can be free to pursue whatever career I want to do, I will still be a community pharmacist at a medicine outlet. | 1 | 2 | 3 | 4 | 5 |

Thank you!

**Vietnamese version**

Mã phiếu: .......................................................

Ngày thu thập dữ liệu: ........./........../...............

Các dược sĩ thân mến. Nhóm nghiên cứu của Trường Đại học Dược Hà Nội đang tiến hành khảo sát nhằm đánh giá mức độ hài lòng với công việc của dược sĩ cộng đồng trong bối cảnh đại dịch COVID-19 tại Việt Nam. Chúng tôi thực sự đánh giá cao sự đóng góp và tham gia của bạn trong nghiên cứu của chúng tôi. Thời gian trả lời câu hỏi khoảng 10 phút. Tất cả thông tin trong biểu mẫu thu thập dữ liệu này chỉ được sử dụng để nghiên cứu. Danh tính của nhà thuốc và dược sĩ sẽ được bảo mật. Chúng tôi hy vọng rằng bạn có thể trả lời một cách đầy đủ và trung thực nhất có thể. Xin chân thành cảm ơn.

Bạn có đồng ý tham gia cuộc khảo sát này không?

🞎 Có 🞎 Không

**Thông tin cơ bản của dược sĩ**

1. Giới tính: 🞎 Nam 🞎 Nữ

2. Năm sinh: .............................................................................................................................................

3. Tình trạng hôn nhân: 🞎 Đã kết hôn 🞎 Chưa kết hôn

4. Trình độ học vấn cao nhất

🞎 Trung cấp 🞎 Cao đẳng

🞎 Đại học 🞎 Sau đại học (Thạc sĩ, Tiến sĩ...)

5. Kinh nghiệm làm việc ở các cửa hàng thuốc (năm): ..............................................................................

7. Thời gian làm việc trong ngày (giờ): ......................................................................................................

8. Lương bình quân hàng tháng (triệu đồng)

🞎 < 2 🞎 2 – dưới 4

🞎 4 – 6 🞎 > 6

9. Số lượng khách hàng trung bình của cửa hàng thuốc mỗi ngày (người)

🞎 < 30 🞎 30 – dưới 50

🞎 50 - 100 🞎 > 100

10. Số điện thoại: ......................................................................................................................................

11. Địa chỉ của cửa hàng thuốc: ................................................................................................................

|  | **Câu hỏi** | **Rất không đồng ý** | **Không đồng ý** | **Bình thường** | **Đồng ý** | **Rất đồng ý** |
| --- | --- | --- | --- | --- | --- | --- |
| **A** | **Hài lòng về điều kiện làm việc, cơ sở vật chất** | | | | | |
| A1 | Tôi hài lòng với cơ sở vật chất tại nhà thuốc. | 1 | 2 | 3 | 4 | 5 |
| A2 | Nơi làm việc khang trang, sạch sẽ, thoáng mát. | 1 | 2 | 3 | 4 | 5 |
| A3 | Tôi được làm việc trong điều kiện an toàn. | 1 | 2 | 3 | 4 | 5 |
| A4 | Nhà thuốc có đủ các trang thiết bị cho công việc chuyên môn (như là máy tính có kết nối mạng Internet, điều hòa...) | 1 | 2 | 3 | 4 | 5 |
| A5 | Nhà thuốc được trang bị đầy đủ các phương tiện giúp phòng chống lây lan dịch bệnh (như khẩu trang, nước rửa tay...) | 1 | 2 | 3 | 4 | 5 |
| **B** | **Hài lòng về bản chất công việc** | | | | | |
| B1 | Tôi hài lòng với vị trí công việc hiện tại. | 1 | 2 | 3 | 4 | 5 |
| B2 | Công việc mà tôi đang làm phù hợp với trình độ chuyên môn và các kĩ năng được đào tạo. | 1 | 2 | 3 | 4 | 5 |
| B3 | Công việc cho phép sử dụng tốt năng lực của tôi. | 1 | 2 | 3 | 4 | 5 |
| B4 | Công việc chuyên môn (như bán thuốc, tư vấn bệnh nhân) đáp ứng được nguyện vọng của tôi. | 1 | 2 | 3 | 4 | 5 |
| B5 | Công việc của tôi được tiếp xúc với nhiều người. | 1 | 2 | 3 | 4 | 5 |
| **C** | **Hài lòng về thu nhập và các quyền lợi khác** | | | | | |
| C1 | Tôi hài lòng với mức thu nhập hiện nay của mình. | 1 | 2 | 3 | 4 | 5 |
| C2 | Tiền lương tôi được trả tương xứng với vị trí công việc. | 1 | 2 | 3 | 4 | 5 |
| C3 | Chế độ thưởng tôi nhận được xứng đáng với hiệu quả làm việc. | 1 | 2 | 3 | 4 | 5 |
| C4 | Cách phân chia thu nhập tăng thêm công bằng. | 1 | 2 | 3 | 4 | 5 |
| **D** | **Hài lòng về người quản lý/công tác quản lý** | | | | | |
| D1 | Người quản lý luôn quan tâm đến đời sống nhân viên. | 1 | 2 | 3 | 4 | 5 |
| D2 | Người quản lý đối xử công bằng và hợp lý với tất cả nhân viên trong nhà thuốc. | 1 | 2 | 3 | 4 | 5 |
| D3 | Người quản lý có đủ năng lực và kiến thức để quản lý hiệu quả các công việc tại nhà thuốc. | 1 | 2 | 3 | 4 | 5 |
| D4 | Người quản lý tin tưởng tôi trong công việc. | 1 | 2 | 3 | 4 | 5 |
| D5 | Người quản lý luôn hỗ trợ, hướng dẫn nhiệt tình nhân viên trong công việc. | 1 | 2 | 3 | 4 | 5 |
| D6 | Người quản lý luôn lắng nghe ý kiến và phản hồi lại nhanh chóng cho nhân viên. | 1 | 2 | 3 | 4 | 5 |
| D7 | Người quản lý luôn khen ngợi/động viên khi nhân viên hoàn thành tốt công việc. | 1 | 2 | 3 | 4 | 5 |
| **E** | **Hài lòng về các mối quan hệ với đồng nghiệp và bệnh nhân/khách hàng** | | | | | |
| E1 | Tất cả đồng nghiệp của tôi đều có năng lực tốt. | 1 | 2 | 3 | 4 | 5 |
| E2 | Đồng nghiệp của tôi rất thân thiện. | 1 | 2 | 3 | 4 | 5 |
| E3 | Đồng nghiệp của tôi thường chia sẻ kinh nghiệm, giúp đỡ nhau trong công việc. | 1 | 2 | 3 | 4 | 5 |
| E4 | Đồng nghiệp của tôi đáng tin cậy. | 1 | 2 | 3 | 4 | 5 |
| E5 | Đồng nghiệp của tôi quan tâm, giúp đỡ nhau trong cuộc sống. | 1 | 2 | 3 | 4 | 5 |
| E6 | Tôi hài lòng với mối quan hệ của tôi với bệnh nhân/khách hàng. | 1 | 2 | 3 | 4 | 5 |
| E7 | Bệnh nhân/khách hàng có thái độ tôn trọng tôi. | 1 | 2 | 3 | 4 | 5 |
| **F** | **Hài lòng về cơ hội học tập và thăng tiến** | | | | | |
| F1 | Tôi hài lòng về việc đào tạo, cơ hội thăng tiến/phát triển của mình tại nhà thuốc. | 1 | 2 | 3 | 4 | 5 |
| F2 | Tôi được nhà thuốc đào tạo về kiến thức, kĩ năng chuyên môn thường xuyên. | 1 | 2 | 3 | 4 | 5 |
| F3 | Nhà thuốc luôn tạo điều kiện cho nhân viên tham gia những khóa tập huấn về chuyên môn (như dược lâm sàng, thông tin và tư vấn sử dụng thuốc, văn bản quản lý dược…). | 1 | 2 | 3 | 4 | 5 |
| F4 | Tôi có nhiều cơ hội thăng tiến lên vị trí cao hơn trong quá trình làm việc khi nỗ lực | 1 | 2 | 3 | 4 | 5 |
| F5 | Tôi có cơ hội học tập và phát huy được khả năng/năng lực của mình trong công việc. | 1 | 2 | 3 | 4 | 5 |
| F6 | Nhà thuốc có chính sách thăng tiến công bằng và rõ ràng. | 1 | 2 | 3 | 4 | 5 |
| **O** | **Kết quả** |  |  |  |  |  |
| O1 | Nhìn chung, tôi hài lòng với công việc hiện tại tại cửa hàng thuốc của mình. | 1 | 2 | 3 | 4 | 5 |
| O2 | Nói chung, tôi muốn gắn bó lâu dài với công việc hiện tại và cơ sở bán thuốc của mình. | 1 | 2 | 3 | 4 | 5 |
| O3 | Nếu tôi có thể tự do theo đuổi bất cứ nghề gì tôi muốn làm, tôi vẫn sẽ là một dược sĩ cộng đồng tại một cửa hàng thuốc. | 1 | 2 | 3 | 4 | 5 |

Xin chân thành cảm ơn.
