# Supplementary material for: An instrument for measuring job satisfaction (VIJS): A validation study for community pharmacists in the context of the COVID-19 pandemic in Vietnam
Source: PLoS One. 2022 Nov 4;17(11):e0276918. doi: 10.1371/journal.pone.0276918 (PMC9635709; doi:10.1371/journal.pone.0276918)
Supplement: Attachment — Submitted filename: Response to Reviewers.docx [file pone.0276918.s003.docx]

**REBUTTAL LETTER**

Dear editors and reviewers.

First and foremost, we want to express our deep gratitude to the editors and reviewers who gave us valuable and practical advice. We carefully read your comments and revised our manuscript. Our responses are written in blue color.

One change in the revised manuscript which I should mention here is our affiliations. In July 2022, our university was re-structured and the names of departments/faculties were changed. The name of our department was also changed from “Department of Pharmaceutical Management and PharmacoEconomics” into “Faculty of Pharmaceutical Management and Economics”.

Thank you so much for your assistance.

Best regards.

Dai Xuan Dinh

Hanoi University of Pharmacy

......................................................................................................

**Journal Requirements**

1. Please ensure that your manuscript meets PLOS ONE's style requirements, including those for file naming. The PLOS ONE style templates can be found at

https://journals.plos.org/plosone/s/file?id=wjVg/PLOSOne_formatting_sample_main_body.pdf and

https://journals.plos.org/plosone/s/file?id=ba62/PLOSOne_formatting_sample_title_authors_affiliations.pdf

We strived to follow these guidelines.

2. Please review your reference list to ensure that it is complete and correct. If you have cited papers that have been retracted, please include the rationale for doing so in the manuscript text, or remove these references and replace them with relevant current references. Any changes to the reference list should be mentioned in the rebuttal letter that accompanies your revised manuscript. If you need to cite a retracted article, indicate the article’s retracted status in the References list and also include a citation and full reference for the retraction notice.

We checked the reference list, revised some references, and deleted one reference: “Barnett CW, Kimberlin CL. Development and validation of an instrument to measure pharmacists' satisfaction with their jobs and careers. Am J Pharm Educ. 1986 Spring;50(1):5-14. PMID: 10281557.”

If there are any references that are inappropriate, please let us know.

3. Please check and correct the entire manuscript carefully in terms of grammar and typography.

For better formatting of the manuscript, the following link contains guidelines for writing the information on the title page and the body of the manuscript. It is suggested to see these guidelines to set different headings in different sections of the manuscript.

We checked all over our manuscript and revised grammar/typographic mistakes.

**Reviewers' comments**

**Reviewer #1:**

The manuscript technically sound, and the data support the conclusions. The statistical analysis has been performed appropriately. The conclusions drawn appropriately based on the data presented. The manuscript presented in clear and comprehensible fashion and written in standard English.

Thank you for your compliments. We did our utmost to conduct this research.

**Reviewer #2:**

This research is very well conducted and i recommend for publication however i would like to make few suggestions.

1. There are few typographic mistakes which need to be corrected all over the manuscript.

We checked all over our manuscript and revised typographic mistakes.

2. VIJS need to be explained more to make it easy for readers and why you select this instrument.

We planned to conduct a study to investigate the job satisfaction of Vietnamese community pharmacists. However, no instruments with Vietnamese versions could be found. As a result, we developed and validated this instrument in phase 1. And then, we will use this instrument to interview community pharmacists all over Vietnam (phase 2).

3. The factors used in different tables also need more explanation.

The factors used in different tables are the same. The VIJS includes the six following factors:

Factor A: Physical working conditions,

Factor B: Work nature,

Factor C: Income and other benefits,

Factor D: Management policies and managers,

Factor E: Relationships with coworkers and customers,

Factor F: Learning and advancement opportunities.

These factors were named after we used EFA with promax rotation. Items for each factor can be seen in Table 1.

4. Figure 2 need explanation.

The explanation of Figure 2 can be seen in lines 189-192. In addition, some relevant information mentioned in above paragraphs (such as information in lines 168-172) was not repeated.
